# Internet- and mobile-based psychological interventions for post-traumatic stress symptoms in youth: a systematic review and meta-analysis

**DOI:** 10.1038/s41746-024-01042-7

**Published:** 2024-02-29

**Authors:** Christina Schulte, Mathias Harrer, Cedric Sachser, Jasmina Weiss, Anna-Carlotta Zarski

**Affiliations:** 1 Professorship Psychology and Digital Mental Health Care, Department Health and Sport Sciences, School of Medicine and Health, Technical University of Munich, Munich, Germany; 2 Department of Child and Adolescent Psychiatry and Psychotherapy, University Ulm, Ulm, Germany; 3 Department of Clinical Psychology, Division of eHealth in Clinical Psychology, Philipps University of Marburg, Marburg, Germany

**Keywords:** Post-traumatic stress disorder, Trauma

## Abstract

Psychological interventions can help reduce posttraumatic stress symptoms (PTSS) in youth, but many do not seek help. Internet- and mobile-based interventions (IMIs) show promise in expanding treatment options. However, the overall evidence on IMIs in reducing PTSS among youth remains unclear. This systematic review and meta-analysis investigated the efficacy of IMIs in PTSS reduction for youth exposed to traumatic events. A comprehensive literature search was conducted in January 2023 including non-randomized and randomized-controlled trials (RCT) investigating the effects of IMIs on PTSS in youth aged ≤25 years. Six studies were identified with five providing data for the meta-analysis. The majority of studies included youth with different types of trauma irrespective of PTSS severity at baseline (*k* = 5). We found a small within-group effect in reducing PTSS from baseline to post-treatment (*g* = −0.39, 95% CrI: −0.67 to −0.11, *k* = 5; *n* = 558; 9 comparisons). No effect emerged when comparing the effect of IMIs to control conditions (*g* = 0.04; 95%-CrI: -0.52 to 0.6, *k* = 3; *n* = 768; *k* = 3; 4 comparisons). Heterogeneity was low between and within studies. All studies showed at least some concerns in terms of risk of bias. Current evidence does not conclusively support the overall efficacy of IMIs in addressing youth PTSS. This review revealed a scarcity of studies investigating IMIs for youth exposed to traumatic events, with most being feasibility studies rather than adequately powered RCTs and lacking a trauma focus. This underscores the demand for more high-quality research.

## Introduction

Epidemiological studies indicate that 56% to 68% of children, adolescents and young adults experience at least one traumatic event by age 16^1–3^. While many youths recover on their own^4^, a considerable proportion suffers from posttraumatic stress symptoms (PTSS) such as intrusive recollections/flashbacks, avoidance of internal and external reminders and hyperarousal^5^. Untreated PTSS can negatively affect social interactions, physical well-being, and educational trajectories^6,7^. Further, they increase the risk of developing mental disorders including major depression, substance abuse or a full-syndrome posttraumatic stress disorder (PTSD)^8–10^. Physiological neurological social, and emotional long-term consequences can persist into adulthood^11,12^.

Guidelines for PTSD from the International Society for Traumatic Stress Studies (ISTSS) recommend preventative interventions within the first three months of a traumatic event to individuals regardless of PTSS or early treatment for individuals with emerging PTSS. For individuals with clinically relevant PTSS or full-syndrome PTSD three months post-trauma, psychological treatment is indicated, especially trauma-focused interventions^13–15^. Trauma focus implies that cognitive, behavioral, or emotional treatment components are used to facilitate processing of the traumatic event(s) as a key part of the therapeutic process^16^. Identified commonalties of different evidence-based manualized treatments are psychoeducation; emotion regulation and coping skills; imaginal exposure; cognitive processing, restructuring, and/or meaning making^17^.

A meta-analysis of 32 randomized controlled trials (RCTs) involving 2.260 youth revealed large effects for individual forms of trauma-focused cognitive behavioral interventions (tf-CBT; *g* = 0.91-2.94) in reducing PTSS post-treatment compared to waitlist conditions^18^. The efficacy of other types of psychological interventions without a trauma focus, e.g., psychoeducational programs or neurofeedback, in treating PTSS in youth has not yet been shown to be effective for recommending their provision^19^. Limited research on the prevention of PTSS in youth includes 27 studies covering various psychological interventions, e.g., psychoeducation, hypnosis and music therapy. These preventive interventions show a trend towards PTSS reduction at individual study level but no conclusion on the overall evidence could be drawn^20^. A meta-analysis of 75 RCTs on PTSD prevention in adults found insufficient evidence to strongly recommend any intervention, except for tf-CBT in cases of acute stress disorder^21^.

In recent years, Internet- and mobile-based interventions (IMIs) have been increasingly studied as an alternative treatment modality, particularly for individuals reluctant to seek traditional help due to barriers like stigma, preferring self-help, and structural shortfalls in health-systems^22–26^. Considering their familiarity with mobile devices, IMIs hold promise to reach youth and overcoming these barriers of attitude, time and place^27,28^. IMIs offer an appealing solution by delivering treatment online more anonymously in a timely and often cost-effective manner with varying levels of guidance^29,30^. Internet-based CBT (i-CBT) has demonstrated moderate effects (*g* = 0.66–0.83) among adults in reducing PTSS at post-treatment compared to waiting list control groups^31,32^ and small effects (*d* = 0.36) compared to active control groups^33^. Increasing evidence suggests that specifically, trauma-focused i-CBT is effective and noninferior to face-to-face tf-CBT^34,35^. Additionally, a study has demonstrated that a trauma-focused i-CBT (iCT-PTSD) was effective and acceptable to patients with PTSD and superior to a non-trauma-focused cognitive behavioral stress management therapy in adults^36^.

In contrast to adults, IMIs for PTSS prevention or treatment in youth have been understudied. There is the need to comprehensively review the current literature and analyze the overall efficacy of IMIs targeting PTSS in youth. Thus, this study aims to systematically review and analyze the efficacy of IMIs in reducing PTSS in trauma-exposed youth.

## Results

### Study selection

**Fig. 1:**
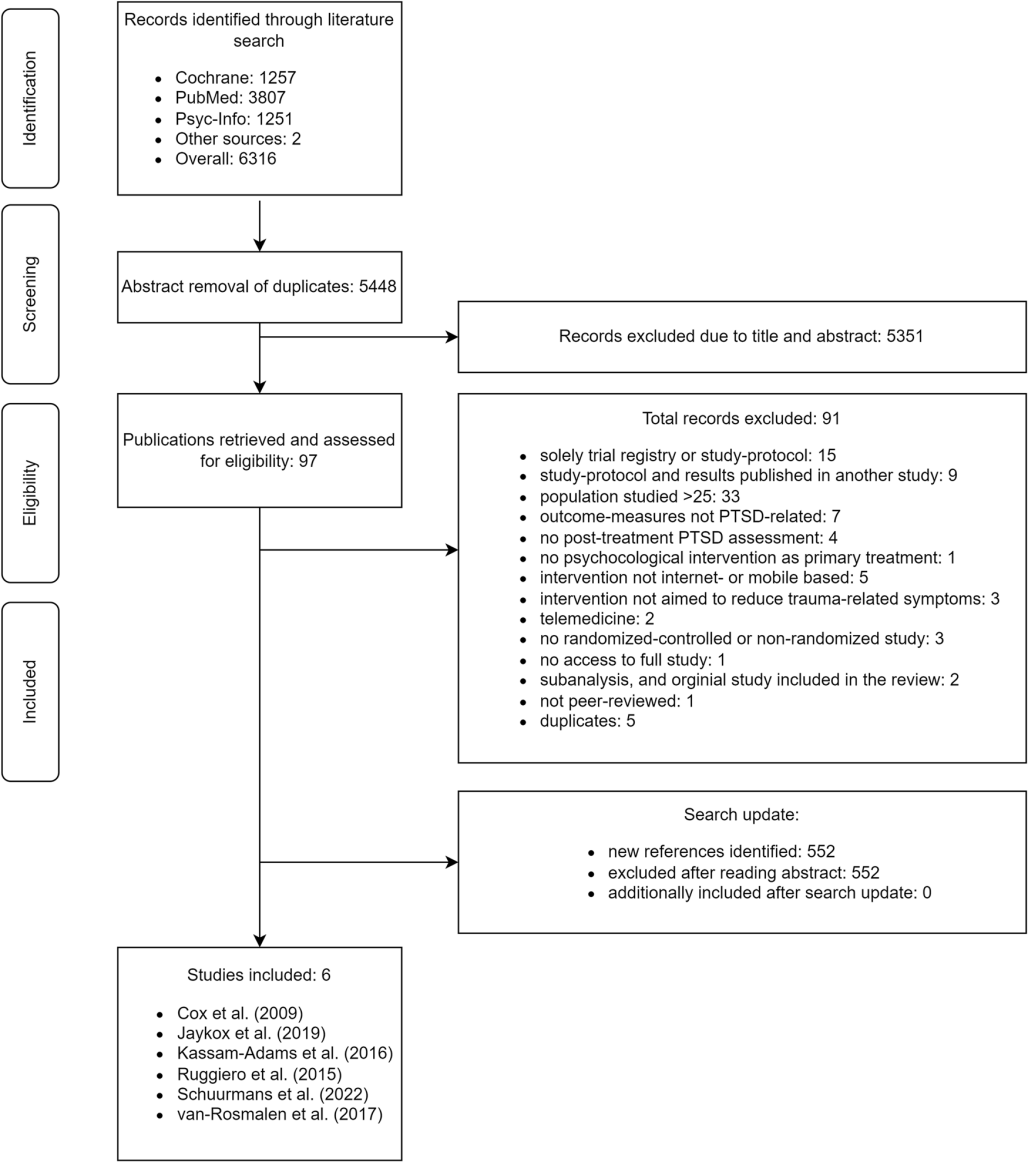
Study flow. PTSD Posttraumatic Stress Disorder.

The electronic database search and manual search conducted on the 26th of January 2023 yielded 6316 records. After removing duplicates, the titles and abstracts of the remaining 5448 records were screened, and 97 articles were included into the full-text screening, resulting in six studies meeting the eligibility criteria. A search update on November 13, 2023 did not identify any further eligible studies for inclusion. For the selection process, interrater-reliability between the two researchers was substantial (Cohen’s Kappa = 0.68)^37,38^. The study selection process and reasons for exclusion are depicted in Fig. 1.

### Studies and intervention characteristics

Table 1 summarizes the study characteristics and Table 2 presents the intervention characteristics. The references of all studies included can be found in Supplementary References.

**Table 1:** Study characteristics

study	country	study design	conditions	*n*, drop-out post	inclusion criteria for study participation	type of trauma	% clinical significant PTSS at baseline	measure (PTSS)	participant age M (SD)	% female
Cox et al. (2009)	AUS	RCT	IMI control (untreated)	*n* = 44, 14% *n* = 41, 14%	children (7–16 y), consenting parents, internet access, hospitalized overnight for unintentional injury	injury	n.i.	TSCC-A (secondary)	10.85 (2.13), 10.95 (2.26)	30 32
Ruggiero et al. (2015)	USA	RCT	IMI IMI + ASH control (PE)	*n* = 270, 26% *n* = 279, 28% *n* = 167, 0%	families with adolescents (12–17 y) residing at household address at time of tornado	natural disaster^a^	8.0% 8.1% 6.7%	NSA-PTSD (primary)	14.49 (1.78), 14.43 (1.83), 14.64 (1.68)	49
Kassam-Adams et al. (2016)	USA, AUS	RCT as feasibility	IMI control (waitlist)	*n* = 36, 33% *n* = 36, 5%	children (8–12 y), wake and aware, recently (2 weeks) admitted to hospital for acute, potentially traumatic medical event, internet access, fluent in English	medical event	61% 39%	CPSS (primary)	9.8 (1.5), 9.8 (1.3)	46
van Rosmalen-Nooijens et al.^46^	NLD	RCT as feasibilty	IMI control (waitlist) IMI (for controls^b^)	*n* = 31, 74% *n* = 26, 65% *n* = 26, 77%	self-registering adolescents (12–25 y), exposed to family violence, fluent in Dutch	family violence	70% 90%	IES (primary)	18.40 (3.62), 18.20 (3.02)	91
Jaycox et al. (2019)	USA	open trial	IMI	*n* = 36, n.i.	middle and high school students (12–19 y)	mixed (death, illness/injury, witnessing violence)	n.i.	CPSS (primary)	n.i.	n.i.
Schuur-mans et al. (2020)	NLD	randomized feasibility	IMI (game version 1) IMI (game version 2) IMI (game version 3)	*n* = 5, 0% *n* = 5, 40% *n* = 5, 40%	adolescents (10–18 y), clinical levels of PTSS (score ≥30 on CRIES-13), living in residential care, consent from legal guardians, fluent in Dutch	n.i.	100% 100% 100%	CRIES-13 (secondary)	14.46 (2.40)	50

*ASH* adult self- help targeting parents’ mental health, *AUS* Australia, *CRIES-13* Children’s Revised Impact of Event Scale^38^, *CPSS* Child PTSD Symptom Scale^39^, *IES* Impact of Events Scale^40^, *USA* United States of America, *n.i.* no information, *NLD* Netherlands, *RCT* randomized controlled trial. *NSA-PTSD* National Survey of Adolescents PTSD module^42^, *PE* psychoeducation, *PTSD* post-traumatic stress disorder, *PTSS* Posttraumatic stress symptoms, *primary PTSS* as primary outcome measure, *SD* standard deviation, *secondary, PTSS* as secondary outcome measures, *TSCC-A* Trauma Symptom Checklist for Children-A^41^, *Y* years.

^a^Tornadoes in Joplin, Missouri, and Alabama in 2011.

^b^Control group with full access to IMI after waiting time.

**Table 2:** Intervention characteristics

study	IMIs name	time from trauma to IMI	PTSS screen for trauma content	therapeutic approach	delivery format	number of modules structure duration	guidance	intervention adherence reminders	intervention adherence measures	PE	coping strategies	cognitive methods	social support	support for on-site service	trauma-focused component
Cox et al. (2009)	Kids and Accidents	≤ 3 months after trauma exposure	unscreened	CT	securewebpage	4 independently^a^ ad libitum	Unguided self-help	n.i.	56% complet.	✓	✓	x	x	x	x
Ruggiero et al. (2015)	Bounce Back Now	> 3 months after trauma exposure	emerging PTSS (3 or more symptoms on NSA-PTSD)	CBT	secure webpage	4 independently^a^ n.i.	self-help	one-time reminder after initial visit	72–75% completed at least one module	✓	✓	x	x	x	✓
Kassam-Adams et al. (2016)	Coping Coach	≤ 3 months after trauma exposure	unscreened	CBT	n.i.	3 sequentially^b^ ~ 1 month	one-time on-site assistance	weekly e-mail reminders	53% complet.	✓	✓	✓	✓	✓	x
van Rosmalen-Nooijens et al.^46^	Feel the Vibe	n.i.	unscreened	PE	secure webpage	n.a. n.a. ad libitum	active support (e.g., moderation) and on demand via website	daily e-mail reminders	61% used IMI ≥ 12 weeks	✓	x	x	✓	✓	x
Jaycox et al. (2019)	LIFT	n.i.	emerging PTSS ( ≥ 10 on CPSS)	CBT	online program via computer in schools	7 sequentially^b^ ~ 7 weeks	self-help	IMI as part of the school program	n.i.	✓	✓	x	x	x	✓
Schuurmans et al. (2020)	Muse DayDream Wild Divine	n.i.	clinically relevant PTSS ( ≥ 30 on CRIES-13)	CBT + neuro-feedback	app-based via iPad or Laptop	12 repetitive^c^ 6 weeks	on-site assistance on demand	RA visiting for module completion	100% complet. 60% complet. 80% complet.	✓	✓	x	x	x	x

*CBT* cognitive behavioral therapy, *CPSS* Child PTSD Symptom Scale^39^, *CRIES-13* Children’s Revised Impact of Event Scale^38^, *CT* cognitive theory, *IMI* Internet- and mobile-based intervention, *LIFT* Life improvements for Teens, *n.a.* not applicable, *n.i.* no information, *NSA-PTSD* PTSD modules of the National Survey of Adolescents PTSD^42^, *PE* Psychoeducation, *PTSS* Posttraumatic Stress Symptoms, *RA* research assistant.

^a^Independently, free selectable content.

^b^sequential, modules built on each other, meaning that in order to move on to the next one, the previous one had to be completed.

^c^repetitive, adolescents always played the same game.

#### Study characteristics

Six studies were identified with four being RCTs, one randomized feasibility study and one open trial. Two of the four RCTs were full clinical studies and the other two RCTs were conceptualized as feasibility studies. The four RCTs included different types of control groups (CG) (untreated control (*k* = 1), waitlist control (*k* = 2), psychoeducation (*k* = 1)). One of the RCTs included two intervention groups (IG) (i.e., IMI for youth; IMI for youth + IMI for parents), and one RCT also evaluated the efficacy of the IMI in the waitlist control group after providing full IMI access after the waiting period (IMI for controls). The randomized feasibility study compared three different versions of an IMI. The open trial was a one-armed trial investigating the IMI in a pre-post design. Five of the six studies provided data on PTSS at post-treatment and were included in the meta-analysis. Three studies with four comparisons evaluated between-group effects of PTSS in *n* = 768 participants at post-treatment (IG: *n* = 533 IG, CG: *n* = 235). Within-group effects in the IG from baseline to post-treatment were available from five studies with nine comparisons (*n* = 558). The six identified studies were published between 2009 and 2019 and were conducted in the United States of America (USA, *k* = 3), Australia (AUS, *k* = 2), and the Netherlands (NLD, *k* = 2), involving a total of *n* = 1379 participants at baseline (*n* range: 15–987). Types of traumatic events participants were exposed to included natural disaster (*k* = 1), injury or medical event (*k* = 2), and family violence (*k* = 1). In one study, the majority of participants (88%) had experienced one or more traumatic event(s); the most prevalent ones included death, illness or injury of a close person, witnessing violence, or being the victim of violence. One study did not provide any information on the type of trauma experienced but included only youth in residential care with clinically relevant PTSS (score ≥ 30 on the Children’s Revised Impact of Event Scale, CRIES^39^). None of the other studies (*k* = 5) required emerging or clinically relevant PTSS for study inclusion. Four studies reported the percentage of participants with clinically relevant PTSS at baseline, ranging from 39% to 100%. The other two studies reported their participants’ symptom levels at baseline to be on average below the cut-off of clinical relevance. PTSS were assessed as the primary outcome in four studies and as secondary outcome in two studies. PTSS were assessed using various scales including the Child PTSD Symptom Scale (CPSS^40^; *k* = 2), the Impact of Events Scale (IES^41^; *k* = 1), the Trauma Symptom Checklist for Children-A (TSCC-A^42^; *k* = 1), the PTSD module of the National Survey of Adolescents (NSA^43^; *k* = 1), and the CRIES (*k* = 1). The mean post-measurement time was nine weeks after baseline (range: 4 to 16 weeks). Five of the six studies reported dropout rates at post-treatment ranging from 0% up to 77%. The mean age of participants ranged from 9.8 to 18.4 years, with gender ratios of 40% to 57% female participants.

#### Intervention characteristics

Two of the six IMIs were provided to individuals within the first three months after trauma exposure, one IMI was provided more than three months (range: 3 to 14 months) after trauma exposure, and the remaining three studies lacked timing details of the IMI in relation to trauma exposure. Two studies screened for presence of PTSS within the IMIs to individually tailor trauma-related content; In one study, elevated PTSS (score ≥ 10 on CPSS) were required to access trauma-related IMI sessions instead of sessions on stressful experiences only, while another IMI encouraged participants with heightened PTSS scores (≥3 on the NSA-PTSD module) to complete all PTSD-related modules. The IMIs were predominantly based on CBT (*k* = 3), or cognitive resilience theory (*k* = 1)^44,45^. One IMI consisted of information-only psychoeducation on PTSS, PTSD, and symptom management (PE, k = 1). Another IMI was a game-based CBT relaxation training together with neurofeedback (CBT+neurofeedback, *k* = 1). Across all IMIs, treatment components comprised psychoeducation (*k* = 6), coping strategies (e.g., relaxation techniques or emotion-regulation strategies, *k* = 3), cognitive methods (*k* = 1), social support (*k* = 2), and finding (regional) treatment services (e.g., on-site psychotherapy, *k* = 2). Two IMIs explicitly addressed the traumatic events via incorporating trauma exposure recommendations (k = 1), or writing a trauma narrative (*k* = 1). Four of the six IMIs had multiple modules (range: 3–7) conducted independently (*k* = 2) or sequentially (*k* = 2). In one IMI, the game-based components remained consistent in terms of content, sequence, and duration over twelve modules and another IMI included different components on a website such as reading tasks and a chat forum. Delivery methods varied, with three IMIs being delivered via a secure webpage, one through computers at school, and one utilizing iPads or laptops. The average duration of the IMIs ranged from one month to open-ended access. Human guidance was included in four IMIs. Guidance incorporated moderation in an online forum (*k* = 1), online-based support on demand (*k* = 1), one-time face-to-face assistance with login and use of the IMI (*k* = 1), and face-to-face support on demand by a research assistant being present while participants worked on the IMI (*k* = 1). Adherence reminders for IMI usage were included in five studies, either in the form of e-mail reminders (*k* = 3) or face-to-face as part of a school program (*k* = 1), or by a research assistant being present for module completion (*k* = 1). Adherence to the IMIs was assessed in five studies, with participant’s IMI completion rates being assessed in two studies (range: 53–100%) and two studies reporting module usage, i.e., number of participants who completed at least one out of four modules, or visited the website for more than twelve weeks.

### Quality of included studies

**Fig. 2:**
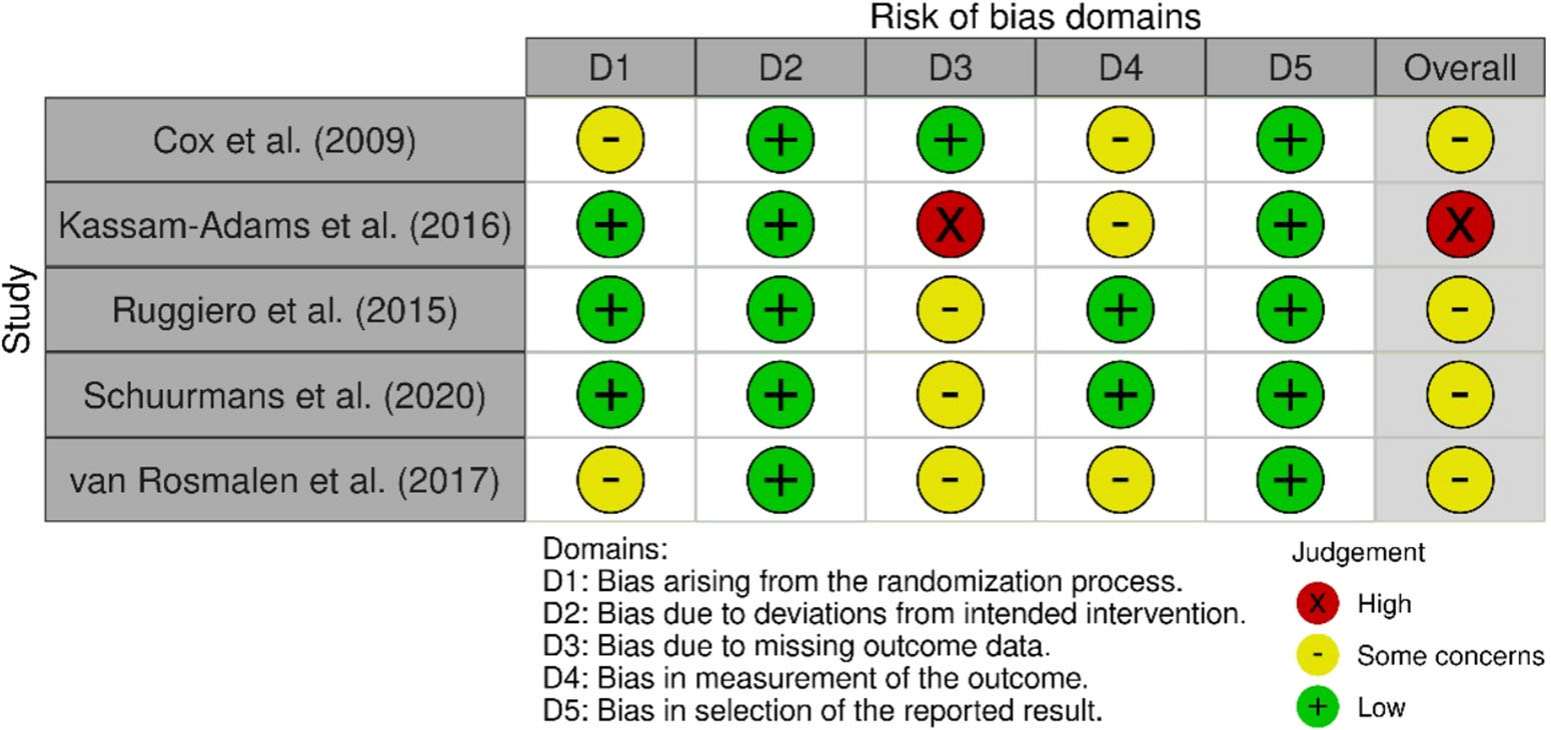
Results for risk of bias rating for randomized studies (*k* = 5) using the RoB 2 tool. red = high risk of bias, yellow = some concerns, green = low risk of bias.

One of the five randomized studies showed an overall high risk of bias (20%), while four studies were classified as raising some concerns (80%). The risk of bias of the studies was classified based on (1) process of randomization (*k* = 3 low, *k* = 2 some concerns), (2) deviations from the intended interventions (*k* = 5 low), (3) missing outcome data (*k* = 1 low, *k* = 3 some concerns, *k* = 1 high), (4) outcome measurement (*k* = 2 low, *k* = 3 some concerns), and (5) selection of reported results (*k* = 5 low) (see Fig. 2). The non-randomized study showed an overall moderate risk of bias (classification by ratings: (1) confounding: low, (2) selection of participants into the study: low, (3) classification of intervention: low, (4) deviations from the intended interventions: low, (5) missing outcome data: moderate, (6) measurement of the outcome: moderate, (7) selection of reported results: moderate).

### Effects of IMIs on PTSS

#### Between-group effects

**Fig. 3:**
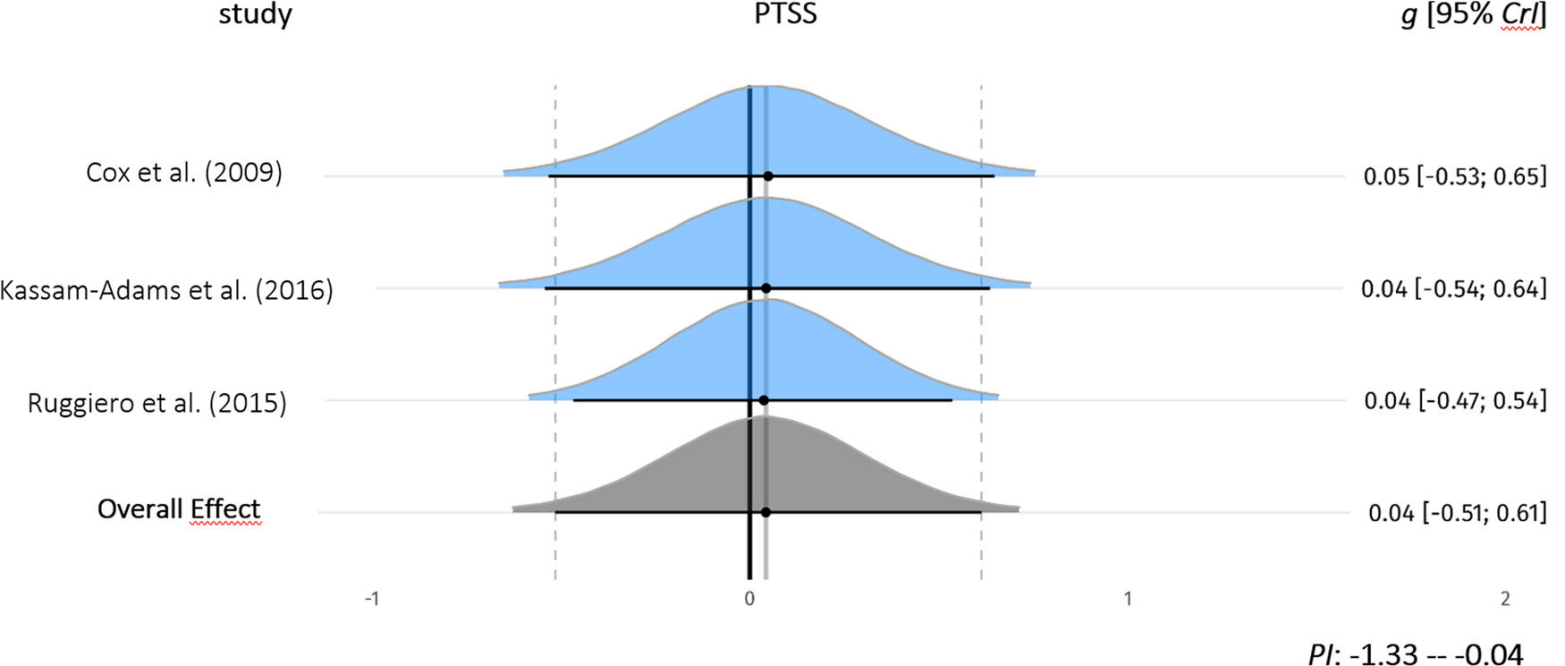
Forest plot for between-group comparisons (*n* = 768) of Posttraumatic Stress Symptoms. Comparisons were untreated control, waitlist control, psychoeducation. *g* = standardized mean difference, Hedges *g*. 95%-CrI = 95% credible interval. *PI* Prediction Interval. Study densities represent the estimated model-based effect, not the empirical values of *g* reported in the original studies. PTSS Posttraumatic Stress Symptoms.

Between-group effects of IMIs on PTSS were investigated in four comparisons from three studies (*n* = 768). One comparison was excluded from the analysis^46^ due to severe baseline imbalances between the study groups for PTSS at baseline [*g* = −0.93]. Due to these severe imbalances, the calculated between-group effect favored the IG at post-test [*g* = −0.82]. Both groups in this trial showed higher PTSS scores at post-treatment compared to baseline indicating worsening of symptoms [IMI (*n* = 8): 31.4 (18.3) at baseline, 37.3 (15.1) at post-assessment; controls (*n* = 9): 47.2 (14.7) at baseline, 49.6 (14.9) at post-assessment)]. For the investigated between-group effect, the 95% credible interval included zero (*g* = 0.04; 95%-CrI: −0.52 to 0.6, *k* = 3). Heterogeneity was low (I^2^ = 7%; 95%-CI: 0–60%) (forest plot Fig. 3), and the prediction interval ranged from *g* = −1.33 to −0.04. We conducted a sensitivity analysis including the study with severe imbalances between the study groups at baseline. Again, the 95% credible interval of the between-group effect included zero (*g* = –0.05; 95%-CrI: −0.57 to 0.3, *k* = 4). Heterogeneity was low (I^2^ = 6%; 95%-CI: 0–51%) and the prediction interval ranged from *g* = −0.89 to −0.99.

#### Within-group effects

**Fig. 4:**
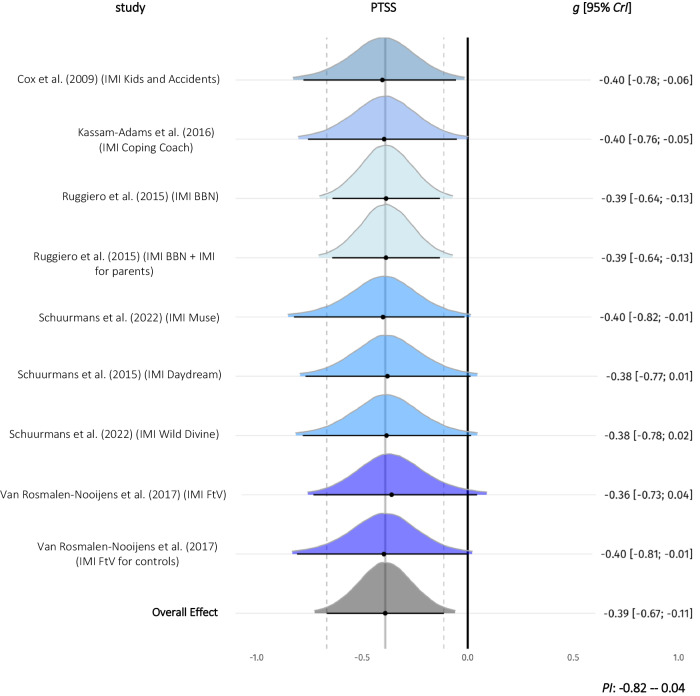
Forest plot for within-group comparisons (*n* = 591) of Posttraumatic Stress Symptoms. *g* = standardized mean difference, Hedges *g*. 95% *CrI* = 95% credible interval. *PI* Prediction Interval. Study densities represent the estimated model-based effect, not the empirical values of *g* reported in the original studies. IMI Internet- and mobile-based Intervention. BBN Bounce Back Now. ASH Adult Self Help. IMI for controls control group with full access to IMI after waiting time. PTSS Posttraumatic Stress Symptoms.

Within-group effects of PTSS were investigated in nine comparisons from five studies (*n* = 591). The overall effect size was small with *g* = −0.39 (*k* = 5) and the 95% credible interval did not include zero (−0.67 to −0.11). Heterogeneity of effects within studies was low (*I*^2^ = 2%; 95%-CI: 0–30%, *k* = 5). Heterogeneity of effects between studies was low (*I*^2^ = 3%; 95%-CI: 0–30%, *k* = 5) with the Prediction Interval ranging from −0.82 to 0.04) (forest plot Fig. 4).

## Discussion

This systematic review and meta-analysis reviewed and analyzed the efficacy of IMIs to reduce PTSS in youth exposed to traumatic events. Six studies were identified including RCTs, feasibility studies and one open trial. The samples involved youth aged ten to 19 years, who have been exposed to different types of traumatic events including interpersonal and non-interpersonal trauma. Most studies did not require increased PTSS severity or PTSD diagnosis for study inclusion, but some studies reported high percentage of individuals with clinical levels of PTSS at baseline. Two studies provided their IMI within the first three months after trauma exposure to unscreened individuals, therefore being preventative. One study provided psychological treatment to youth with clinical significant PTSS. The majority of IMIs was based on CBT, without incorporating trauma-focused intervention components. The meta-analytic results showed a small within-group effect size (*g* = −0.39) for IMIs in reducing PTSS from baseline to post-treatment. No between-group effects on PTSS were observed. Heterogeneity was low for effects between and within studies. At least some concerns regarding risk of bias were present in all studies.

The limited number of identified studies underscores the scarcity of research on IMIs for youth with PTSS, in particular in contrast to adults. In adults, the most recent meta-analysis identified 33 randomized controlled trials of internet-based cognitive-behavioral therapies (i-CBTs) for PTSD^33^, highlighting the contrast in the available research between these two age groups. The disparity between the extent of research on IMIs in youth and adults is also reported for other mental health disorders like depression and anxiety^47^. Though the precise causes of this disparity in scientific research remain uncertain, ethical, legal, and practical requirements such as the involvement of caregivers could potentially play a role. Especially for younger children with trauma exposure, the active engagement of caregivers is recommended due to its positive effect on youth recovery^48^, which increases complexity in IMIs. Dyadic eHealth interventions for children and their supporting parents have, however, shown promising effects regarding feasibility and efficacy for different mental disorders^49^.

The small effects of IMIs for PTSS in youth found in this meta-analysis are considerably lower compared to those in meta-analytic studies for face-to-face tf-CBT in youth. The latter showed medium to large between-group effect sizes (*g* = 0.52–2.94) compared to various control conditions (e.g., waitlist, treatment as usual)^18,50^. Additionally, the effects observed in this study are also notably lower when contrasted with those found for adult i-CBT, where moderate to large between group effect sizes have been reported compared to passive waitlist control groups (*g* = 0.71–0.83)^31,32^, and even active control groups with small effect sizes (*g* = 0.38)^33^. One explanation for this divergence could be that the meta-analyses on tf-CBTs and i-CBTs only included studies on PTSD treatment. Hence, participants reported at least emerging PTSS symptoms, while most studies in this review did not require emerging PTSS levels at baseline. When compared to the results of meta-analytic studies on preventative interventions for adults^21^, the results of this review align that there is currently no evidence for efficacy of any preventative intervention. Another explanation for the small within-group effects and the lack of between-group effects could be that the majority of IMIs did not incorporate trauma-focused treatment components. Although two IMIs integrated a component explicitly addressing the traumatic event(s), none of these components was comparable in terms of intensity, duration, and content with trauma-focused treatment components from face-to-face tf-CBT or i-CBT (e.g., recommendations for exposure instead of comprehensive psychoeducation and clear instructions for imaginal exposure)^15,19^. I-CBTs for adults with PTSS for example often involves multiple writing tasks focusing on the traumatic event and/or its consequences within the online-session or to be conducted independently at home and to be reread multiple times^51,52^. Furthermore, the robustness of our between-group estimates is limited due to the restricted number of identified randomized controlled trials contributing to the analysis.

All studies were rated with at least some concerns regarding risk of bias, primarily due to missing outcome data or measurement of the outcome. The prevalence of missing data is a common issue in psychological intervention studies in general^53,54^, and particularly in internet-based interventions in youth, where an average drop-out rate to study-assessments of 25% was found^55^. Accurate identification of intervention effects hinges on proper handling of missing data using appropriate missing data treatments^53^. Unfortunately, some studies failed to adequately report their rates of missing data, their strategies for addressing them, or employed outdated methods. Consequently, it is imperative for future studies to transparently report missing data and employ modern methods to reduce risk of bias^56^. In terms of the risk of bias related to outcome measurement, many of the studies utilized self-report measures. In this case, blinding regarding the received intervention is not possible, and it cannot be ruled out that the outcome assessment may have been influenced by knowledge of the intervention received. To mitigate this risk of bias, studies should include observer-based assessments conducted by assessors who are blinded to the intervention.

Several limitations have to be considered when interpreting the present findings. First, the overall number of studies and comparisons included in the review was small. Although we conducted an extensive search, we cannot exclude the possibility that additional studies meeting the eligibility criteria exist. Second, most of the studies included small sample sizes and appeared to be feasibility studies. Therefore, they were not designed and powered to assess efficacy, which limits the possibility to identify small effects. Third, the small number of included studies in this review and meta-analysis did not allow us to determine to what extent publication bias could be presumed. Fourth, we investigated preventative and (early) treatment interventions jointly, thus no conclusions can be drawn regarding the respective approaches. Fifth, for a comprehensive overview of IMIs in youth trauma research, we employed a broad inclusion criterion, encompassing studies targeting the reduction of trauma-related symptoms or PTSS. This led to the evaluation of diverse study populations, from trauma-exposed individuals without acute symptoms to those clinically diagnosed with PTSD, limiting the generalizability of results to a specific population. Sixth, vague intervention descriptions led to difficulties in the inclusion or exclusion of studies, resulting in more frequent discussions to resolve discrepancies and a decrease in inter-rater reliability. Seventh, no subgroup analyses could be performed, due to the small sample sizes of included studies. Eighth, meta-analytic within-group effects have to be interpreted with caution, as baseline and post-treatment data are not independent from each other, potentially leading to substantial error in the estimation of effects^57^. Ninth, the overall risk of bias for the studies reviewed was rated with some concern for the majority of studies and one study displayed high risk of bias, limiting the reliability of findings.

Given that many youths encounter barriers in seeking face-to-face support and express a preference for self-help^23^, exploring the potential of IMIs for youth and capitalizing on their benefits is important. By presenting the current evidence, we seek to stimulate research interest, prompt future studies to fill gaps, and guide researchers in the evolving landscape of IMIs for youth with traumatic experiences, which is especially important in areas with limited evidence. Despite acknowledging limitations related to the small number of included studies, the results of this study add value to research due to a robust search strategy, meticulous screening, and methodological rigor. Further high-quality studies are necessary to establish the feasibility and efficacy of IMIs for youth exposed to traumatic events. Concerning the lack of available research in this field, it would be valuable to investigate the barriers that might hinder researchers from studying IMIs in youth with PTSS and develop strategies to address them. Moreover, future studies on IMIs for youth exposed to traumatic events should more clearly distinguish between preventative and (early) treatment interventions. Here, it would be useful to align with prevention and treatment guidelines and define inclusion criteria accordingly. Concerning the diagnostic and assessment, it would be valuable to conduct clinical (semi-) standardized interviews to assess PTSD diagnosis or level of PTSS at baseline. As trauma-focused interventions have been shown to be particularly effective in treating emerging or clinically relevant PTSS in youth^13^, IMIs incorporating a trauma focus should be developed and studied. In this context, it is crucial for future research to clearly describe the intervention used to ensure a clear categorization and understanding of the intervention components. It should be investigated how effective face-to-face tf-CBT for youth and trauma-focused i-CBTs in adults can be translated and implemented in the online context serving youth with PTSS. For this reason, it seems useful to explore the needs and wishes of affected young people for IMIs and to involve them in a co-design approach^58^. Furthermore, research should review the literature on blended approaches that integrate internet-based interventions and face-to-face sessions for treating PTSD. Such approaches show promise, as they are currently primarily studied in adult veterans^59^.

In this systematic review and meta-analysis, six studies on IMIs for youth exposed to traumatic events have been identified displaying a significant small within-group effect (*g* = −0.39) and no between-group effect in reducing PTSS from baseline to post-treatment. Most studies did not include trauma-focused intervention components and were not directed at individuals with heightened PTSS levels, which may have hampered the effects. Given the small number of available studies, the results highlight the need for more high-quality research. The current evidence is insufficient to draw conclusions regarding the overall efficacy of IMIs for PTSS in youth. No between-group effects were observed, but small within-group effects were found, suggesting initial indications of potential efficacy. Due to the limited number of studies and participants, primarily comprising feasibility studies rather than adequately powered RCTs, further development and investigation of IMI-based approaches in youth are warranted.

## Methods

### Registration

This review was preregistered on the 24th of January 2023 under the *Open Science Framework* (OSF, registration 10.17605/OSF.IO/AJMBC), and was conducted according to the Preferred Reporting Items for Systematic and Meta-analysis (PRISMA) guidelines and the Cochrane Handbook for Systematic Reviews of Interventions^60–62^.

### Information sources and search strategy

Between January 24th and 26th, 2023, we conducted searches on the PsychInfo, PubMed, Cochrane Database of Systematic Reviews, and Cochrane Central Register of Controlled Trials databases following the pre-registration of the study. The results of all searches were not limited to a specific timeframe. Search terms for trauma-related symptoms (e.g., PTSD, trauma, posttraumatic), delivery method (e.g., online, internet, computer), type of therapy (e.g., psychological, psychotherapy, intervention) and for age group (e.g., adolescents, young adults, youth) were used, with appropriate adaptations for each database (see Supplementary Tables 1, 2 and 3). Additional sources were identified via organic backward (via reference list search) and forward (citation search) searches.

### Eligibility criteria

To be considered for the review, the studies had to fulfill the following eligibility criteria: (1) peer-reviewed, (2) randomized-controlled and non-randomized studies using (3) a computer-, internet- or mobile-based (4) psychological intervention as the primary treatment, (5) aiming to reduce trauma-related symptoms or PTSS (measured via standardized diagnostic interviews or validated self-report measures) (6) at post treatment, with (7) a PTSD symptom-related measure as primary or secondary outcome (PTSD symptom severity or frequency), in (8) children, adolescents, and young adults aged ≤25 years. The choice of age cut-off was based on the United Nations’ definition of the developmental stages from childhood to young adulthood, whereby “youths” are defined as individuals aged between 15 and 24. Notably, this definition is often construed to include individuals up to the completion of their 25th year of life^46^. Consequently, we opted to establish the age cutoff at 25 years. Studies were excluded from the analysis, if they evaluated (9) blended interventions (i.e., a combination of face-to-face and online sessions), (10) treatments in which the internet was used by therapists and patients for communication only, (11) telemedicine, or (12) interventions that were equivalent in structure and implementation to face-to-face therapy (e.g., therapy via video). No language restrictions were applied apart from formulating the search string in English.

### Selection process and data extraction

Study selection began with screening of the title and abstracts of eligible studies identified in the search. Then, the studies were retrieved and assessed through full-text analysis. Both steps were conducted by two independent reviewers (ChS and JW). Any discrepancies in the ratings were solved in mutual consent, or with referring to a third reviewer (ACZ). The following data were extracted from the studies, if applicable: (1) Bibliographical data (i.e., author(s), title, year), (2) study design features (i.e., study type, controls, sample size, inclusion criteria, outcome measures), (3) sample characteristics (i.e., type of trauma, age, gender ratio), (4) intervention characteristics (i.e., name, time from trauma to IMI, PTSS screening for trauma-related content, therapeutic approach, components, guidance, duration, number of modules, delivery format, adherence) and (5) data required to calculate within-group effect sizes (*means* and *standard deviations* of PTSS measure at baseline and at post-treatment for intervention groups), or between-group effect sizes for RCTs (*means* and *standard deviations* of PTSS measure at post-treatment, for intervention and control groups). In case of missing information, authors of the original studies were contacted. In case of non-response of contacted authors, or when the information provided was insufficient to perform a meta-analysis, the respective articles were excluded from the statistical analysis.

### Data analysis

The revised Cochrane Risk of Bias assessment tool 2^63^ was used for examining the quality of included randomized studies. For non-randomized studies the Risk Of Bias In Non-randomized Studies of Interventions tool (ROBINS-I^64^; was applied. Risk of bias assessment was conducted by two researchers (ChS and JW) considering the information provided in each article. In case of insufficient information in the original articles, related study protocols or preregistrations were identified for assessment. Risk of bias was rated according to the guidelines and related decision trees for each randomized study, as either “low”, raising “some concerns”, or “high”. For non-randomized studies, the Risk of Bias was rated as either “low”, “moderate”, “serious”, or “critical”.

### Statistical analysis

A meta-analysis was performed using R version 4.2.0^65^ and JAGS version 4.3.0^66^ and a narrative method was used to categorize the extracted data. For each study, we calculated the small sample bias-corrected standardized mean difference (Hedges’ *g*) between pre- and post-test scores per group. Mean differences were standardized by the pre-test standard deviation^67^. We assumed a correlation of $\rho$= 0.8 to calculate the standard error^68^. For randomized controlled trials, we also calculated the value of *g* comparing the intervention and control group means at post-test. For studies with multiple intervention groups, we implemented a Bayesian three-level hierarchical model to accommodate the nested data structure (effect sizes in trials). The 95% credible interval (95%CrI), in which the true average effect is located with 95%, was used to confirm the presence of an effect (i.e., if the 95%CrI did not include zero, we concluded that there is a true effect). A weakly informative Half-Cauchy prior ${\mathscr{H}}{\mathscr{C}}$ (0, 0.04) was used to determine the between-study heterogeneity variance (components) *τ*^2^. Such weakly informative priors have been found to have desirable properties particularly when the number of included effect sizes is low^69^, as was the case in this study. Flat ${\mathscr{N}}$(0, 1 × 10^5^) priors were used for the true average effect µ and for all other model parameters. Prediction intervals were calculated around the pooled effect estimates to indicate the range in which the effect of new studies is expected to fall based on present evidence. Following present recommendations in the methodological literature^67,70^ due to the small number of studies included, we did not evaluate publication bias or conduct subgroup analyses. All code and data used to conduct the analyses is publically available on Github (github.com/mathiasharrer/meta-ptsd-adol). A versioned release has been registered with Zenodo (10.5281/zenodo.8246028).

### Reporting summary

Further information on research design is available in the Nature Research Reporting Summary linked to this article.

### Supplementary information

Supplemental Material

Reporting Summary

## Data Availability

Data collected and used in this meta-analysis can be requested from the corresponding author. The underlying code for this study is publically available on Github (github.com/mathiasharrer/meta-ptsd-adol). A versioned release has been registered with Zenodo (doi.org/10.5281/zenodo.8246028).
