# Supplementary material for: Internet- and mobile-based psychological interventions for post-traumatic stress symptoms in youth: a systematic review and meta-analysis
Source: NPJ Digit Med. 2024 Feb 29;7:50. doi: 10.1038/s41746-024-01042-7 (PMC10904807; doi:10.1038/s41746-024-01042-7)
Supplement: Supplementary file 1 — Supplemental Material [file 41746_2024_1042_MOESM1_ESM.docx]

# Supplementary Information

## Supplementary Table 1

Search String entered into PubMed for the Systematic Literature Search.

| Line 1  Youth | ("adolescent"[MeSH Terms] *OR* "young adult"[MeSH Terms] *OR* "adolesc*"[Title/Abstract] *OR* "teen*"[Title/Abstract] *OR* "preteen*"[Title/Abstract] *OR* "youth*"[Title/Abstract] *OR* "young adult*"[Title/Abstract] *OR* "child*"[Title/Abstract] *OR* "minor*"[Title/Abstract] *OR* "juven*"[Title/Abstract] *OR* "young people"[Title/Abstract]) |
| --- | --- |
|  | AND |
| Line 2  Traumatic exposure | ("stress disorders, traumatic"[MeSH Terms] *OR* "stress disorders, post traumatic"[MeSH Terms] *OR* "trauma*"[Title/Abstract] *OR* "posttraumatic*"[Title/Abstract] *OR* "post traumatic*"[Title/Abstract] *OR* "ptsd*"[Title/Abstract] *OR* "cptsd*"[Title/Abstract]) |
|  | AND |
| Line 3  Internet- and mobile based psychological intervention | ("internet"[MeSH Terms] *OR* "internet based intervention"[MeSH Terms] *OR* "telemedicine"[MeSH Terms] OR "therapy, computer assisted"[MeSH Terms] *OR* "internet*"[Title/Abstract] *OR* "tele*"[Title/Abstract] *OR* "online"[Title/Abstract] *OR* "computer"[Title/Abstract] *OR* "digital*"[Title/Abstract] *OR* "virtual*"[Title/Abstract] *OR* "mobile*"[Title/Abstract] *OR* "cyber*"[Title/Abstract] *OR* "phone*"[Title/Abstract] *OR* "icbt"[Title/Abstract] *OR* "ccbt"[Title/Abstract] *OR* "e-therapy"[Title/Abstract] *OR* "etherapy"[Title/Abstract] *OR* "ehealth"[Title/Abstract] *OR* "e-health"[Title/Abstract] *OR* "web-based"[Title/Abstract] *OR* "webbased"[Title/Abstract] *OR* "smartphone"[Title/Abstract] *OR* "health app"[Title/Abstract] *OR* "electronic*"[Title/Abstract])  AND  ("mental health"[MeSH Terms] *OR* "adolescent health"[MeSH Terms] *OR* "mental health recovery"[MeSH Terms] *OR* "therap*"[Title/Abstract] *OR* "psychotherap*"[Title/Abstract] *OR* "treatment*"[Title/Abstract] *OR* "intervention*"[Title/Abstract] *OR* "self-help"[Title/Abstract] *OR* "health promotion"[Title/Abstract]) |

## Supplementary Table 2

Search String entered into Cochrane for the Systematic Literature Search.

| # 1  Youth | [mh Adolescent] OR [mh "Young Adult"] OR ((adolesc*) OR (teen*) OR (preteen*) OR (youth*) OR (“Young NEXT adult”) OR (child*) OR (minor*) OR (juven*) OR (“young people”)):ti,ab  AND |
| --- | --- |
| # 2  Traumatic exposure | [mh "Stress Disorders, Traumatic"] OR [mh "Stress Disorders, Post-Traumatic"] OR ((trauma*) OR (posttraumatic*) OR (post-traumatic*) OR (PTSD) OR (cPTSD)):ti,ab  AND |
| # 3  Internet- and mobile-based psychological interventions | [mh Internet] OR [mh Telemedicine] OR [mh “Therapy, Computer-Assisted”] OR [mh "Internet-Based Intervention"]  OR ((tele*) OR (icbt) OR (ccbt) OR (e-therap*) OR (etherap*) OR (ehealth) OR (e-health) OR (teletherap*)):ti,ab OR (((internet-based) OR (internet-delivered) OR (internet*) OR (electronic) OR (online*) OR (computer*) OR (web-based) OR (digital*) OR (virtual*) OR (mobile*) OR (smartphone) OR (email) OR (e-mail) OR (app) OR (application) OR (cyber) OR (phone) OR (tele*))  AND  ((care*) OR (therap*) OR (psychotherap*) OR (treatment*) OR (intervention*) OR (self-help) OR (selfhelp) OR (counseling) OR (consultation) OR (rehabilitation) OR (support*) OR (“health promotion”))):ti,ab |

## Supplementary Table 3

Search String entered into PsycINFO for the Systematic Literature Search.

| Line 1  Youth | (DE Adolescent Development OR DE Emerging Adulthood OR TI (Adolesc* OR Teen* OR Preteen* OR Youth* OR "Young adult*" OR child* OR minor* OR juven* OR "young people") OR AB (Adolesc* OR Teen* OR Preteen* OR Youth* OR "Young adult*" OR child* OR minor* OR juven* OR "young people"))  AND |
| --- | --- |
|  |  |
| Line 2  Traumatic exposure | ((DE ("Stress and Trauma Related Disorders" *OR* "Trauma" *OR* "Posttraumatic Stress " *OR* "Posttraumatic Stress Disorder " *OR* "Complex PTSD" *OR* " Emotional Trauma" *OR* " Trauma Treatment" *OR* " Traumatic Experiences") *OR* TI (posttraumatic* *OR* post-traumatic* *OR* PTSD *OR* cPTSD) *OR* (AB posttraumatic* OR post-traumatic* *OR* PTSD *OR* cPTSD))  AND |
|  |  |
| Line 3  Internet- and mobile-based psychological intervention | ((DE ("Online Therapy" *OR* "Digital Interventions" *OR* "Telemedicine" *OR* "Telepsychology" *OR* "Electronic Health Services" *OR* "Mobile Health") *OR* TI (internet* *OR* tele* *OR* online* *OR* computer* *OR* digital* *OR* virtual* *OR* mobile* *OR* cyber* *OR* phone* *OR* icbt *OR* ccbt *OR* e-therapy *OR* etherapy *OR* ehealth *OR* e-health *OR* web-based *OR* webbased *OR* smartphone *OR* “health app” *OR* application* *OR* electronic) *OR* AB (internet* *OR* tele* *OR* online* *OR* computer* *OR* digital* *OR* virtual* *OR* mobile* *OR* cyber* *OR* phone* *OR* icbt *OR* ccbt *OR* e-therapy *OR* etherapy *OR* ehealth *OR* e-health *OR* web-based *OR* webbased *OR* smartphone *OR* “health app” *OR* application* *OR* electronic))  AND  ((DE ("Mental Health" *OR* "Adolescent Health" *OR* "Mental Health Services") *OR* TI (care* *OR* therapy* *OR* psychotherap* *OR* treatment* *OR* intervention* *OR* self-help *OR* selfhelp *OR* counseling *OR* consultation *OR* rehabilitation *OR* support *OR* “health promotion”) *OR* AB (care* *OR* therapy* *OR* psychotherap* *OR* treatment* *OR* intervention* *OR* self-help *OR* selfhelp *OR* counseling *OR* consultation *OR* rehabilitation *OR* support *OR* “health promotion”)) |

# Supplementary References

References of included studies.

Cox, C. M., Kenardy, J. A., & Hendrikz, J. K. (2009). A randomized controlled trial of a web-based early intervention for children and their parents following unintentional injury. *Journal of pediatric psychology, 35*(6), 581-592.

Jaycox, L. H., Ayer, L., Vona, P., Hehman, C., Stein, B. D., Mahmud, A., Woolley, M., Meza, E., Thornton, E., & Venkatesh, B. (2019). Development and preliminary evaluation of a self-guided, internet-based tool for coping with stress and trauma: Life Improvement for Teens (LIFT). *Psychological services*, *16*(1), 85–94. https://doi.org/10.1037/ser0000277

Kassam-Adams, N., Marsac, M. L., Kohser, K. L., Kenardy, J., March, S., & Winston, F. K. (2016). Pilot randomized controlled trial of a novel web-based intervention to prevent posttraumatic stress in children following medical events. *Journal of pediatric psychology, 41*(1), 138-148.

Ruggiero, K. J., Price, M., Adams, Z., Stauffacher, K., McCauley, J., Danielson, C. K., Knapp, R., Hanson, R. F., Davidson, T. M., Amstadter, A. B., Carpenter, M. J., Saunders, B. E., Kilpatrick, D. G., Resnick, H. S. (2015). Web intervention for adolescents affected by disaster: Population-based randomized controlled trial. *Journal of the American Academy of Child & Adolescent Psychiatry, 54*(9), 709-717.

Schuurmans, A. A., Nijhof, K. S., Scholte, R., Popma, A., & Otten, R. (2020). A novel approach to improve stress regulation among traumatized youth in residential care: Feasibility study testing three game‐based meditation interventions. *Early Intervention in Psychiatry, 14*(4), 476-485.

van Rosmalen-Nooijens, K., Lo Fo Wong, S., Prins, J., & Lagro-Janssen, T. (2017). Young people, adult worries: randomized controlled trial and feasibility study of the internet-based self-support method “Feel the ViBe” for adolescents and young adults exposed to family violence. *Journal of medical internet research, 19*(6), Article e204.
